# PROTOCOL: Treatment for depressive disorder among adults: An evidence and gap map of systematic reviews

**DOI:** 10.1002/cl2.1308

**Published:** 2023-03-05

**Authors:** Liping Guo, Jieyun Li, Howard White, Zheng Xu, Junjie Ren, Xinyu Huang, Yaogeng Chen, Kehu Yang

**Affiliations:** ^1^ Evidence‐Based Medicine Center, The Centre of Evidence‐based Social Science, School of Basic Medicine Lanzhou University Lanzhou China; ^2^ The Centre of Evidence‐based Social Science, School of Public health Lanzhou University Lanzhou China; ^3^ School of Basic Medicine Ningxia Medical University Ningxia China

## Abstract

This is the protocol for a Campbell evidence and gap map. The objective of the map is to map available systematic reviews on the effectiveness of treatments for depressive disorders among adults. Specifically, this EGM includes studies on the effectiveness of treatments across a range of outcome domains.

## 1 BACKGROUND

### 1.1 Introduction

#### 1.1.1 The problem, condition, or issue

Depressive disorder is characterized as feeling sad or having a depressed mood, having feelings of worthlessness or guilt, experiencing loss of interest, pleasure, or energy, having increased fatigue and lack of physical activity, and having difficulty thinking, concentrating, or making decisions (American Psychiatric Association, 2013; World Health Organization, 1993).

Depression is a common mental disorder affecting 3.8% of the population (approximately 280 million people) and 5.0% of adults worldwide (Institute of Health Metrics and Evaluation, 2021). It accounted for over 50 million Years Lived with Disability (YLD) in 2015. More than 80% of this mental burden occurred in low‐ and middle‐income countries (World Health Organization, 2017). This disorder can be long‐lasting or recurrent and can dramatically affect a person's ability to function and live a rewarding life. It is the single largest contributor to non‐fatal health loss (7.5% of all YLD) and suicide globally (Jacob, 2014; World Health Organization, 2017).

According to the fifth edition of the Diagnostic and Statistical Manual of Mental Disorders (DSM‐V), depressive disorders include disruptive mood dysregulation disorder, major depressive disorder (including major depressive episodes), persistent depressive disorder (dysthymia), premenstrual dysphoric disorder, substance‐ and medication‐induced depressive disorder, depressive disorder due to another medical condition, other specified depressive disorder, and unspecified depressive disorder (American Psychiatric Association, 2013). As disruptive mood dysregulation disorder is more frequent among children and adolescents than adults, it is not considered in the present study, whereas the other depressive symptoms will be included. Although bipolar depressive disorder was separated from depressive disorders in DSM‐V, it will also be included in the present review due to its high frequency among adults (American Psychiatric Association, 2013).

#### 1.1.2 The intervention

Depressive disorders have attracted worldwide attention (Belkin, 2016; Saxena, 2019), and many programs have been developed to prevent this mental health crisis, such as Manochaitanya (Manjunatha, 2016), President's Program (Mirza, 2019), and the mental health Gap Action Program developed by the World Health Organization. The recommendations for the general adult population provided by the APA Guideline Development Panel identified three types of treatment for initial or relapse depression: psychotherapy, pharmacotherapy, and complementary and alternative treatments (American Psychological Association, 2019). In more detail:

- Psychotherapy is any psychological service provided by a trained professional that primarily uses forms of communication and interaction to assess, diagnose, and treat dysfunctional emotional reactions, ways of thinking, and behavior patterns. Psychotherapy may be provided to individuals, couples, families, or members of a group (American Psychological Association, 2015). There are many types of psychotherapy. The main approaches generally include behavioral therapy, cognitive therapy (including cognitive, cognitive‐behavioral, and mindfulness‐based therapy), interpersonal psychotherapy, psychodynamic therapy, and supportive therapy (American Psychological Association, 2019).
- Pharmacotherapy refers to the use of pharmacological agents in the treatment of mental disorders (American Psychological Association, 2015), and second‐generation antidepressants such as venlafaxine, trazodone, bupropion, and mirtazapine are recommended in the guideline (American Psychological Association, 2019).
- Complementary and alternative treatments, also called complementary and alternative medicine (CAM), are a group of therapies and healthcare systems that fall outside the realm of conventional western medical practice. These include but are not limited to acupuncture, chiropractic, meditation, aromatherapy, homeopathy, naturopathy, osteopathy, touch therapy, reflexology, reiki, and the use of certain dietary supplements (American Psychological Association, 2015).

There are many treatments for depression, but more than 75% of adults from low‐ and middle‐income countries receive no treatment (Evans‐Lacko, 2018). Furthermore, evidence regarding the effectiveness of interventions is not uniform, covering different intervention types, settings, and target populations and making it difficult to navigate (Gabriel, 2022; Lee, 2022; Wickersham, 2022). Therefore, this study aims to produce an evidence and gap map (EGM) of systematic reviews, based on the Campbell Guidelines of EGMs (White, 2020). Specifically, this study seeks to produce an EGM of systematic reviews concerned with interventions for the treatment of depressive disorders. This EGM is designed to collect and display all systematic reviews that reported the effectiveness of interventions to treat depressive disorder to inform research commissioning and provide a scientific basis for the development of healthcare policies and practices.

#### 1.1.3 Why it is important to develop the EGM

Although the American Psychiatric Association (APA) had released guidelines for the treatments of depression (American Psychological Association, 2019), not all interventions were considered, particularly in the era of rapid development of science and technology. For instance, several new therapies have emerged that may be a better choice for some patients, such as animal‐assisted therapy (Majić, 2013), virtual reality exposure therapy (Baghaei, 2021), and e‐mental health apps (Porras‐Segovia, 2020). However, updating guidelines is time‐consuming and costly. Moreover, the “best treatment” is not only determined by effectiveness but also by resource availability, patient preferences, and doctors' experience. Hence, whilst waiting for updated guidelines an EGM is a useful starting point for decision‐makers and members of the public seeking evidence‐based advice.

The EGM is a table that offers a visual presentation of the available evidence for a particular sector. The map provides an overview of what studies are available but does not summarize the findings. It is a decision‐making and research priority‐setting tool that highlights gaps in research and provides information for strategic health and social policies, programs, and research priorities (Saran, 2020; White, 2020). It is estimated that 85% of research investment is wasted, and some of these problems can be avoided by prioritizing research, including using systematic reviews (SRs) to rigorously evaluate available evidence before funding or conducting new research (Chalmers, 2014). EGMs can be used to avoid unnecessary duplication and determine whether there is sufficient evidence on which to base decisions or sufficient research for knowledge synthesis (Snilstveit, 2016).

#### 1.1.4 Existing EGMs

We searched the Campbell Library, Cochrane Library, PubMed, EPPI, and 3ie (International Initiative for Impact Evaluation) and identified two EGMs related to the treatment of depression (Britton, 2021; Campisi, 2020). Campisi et al. (2020) created an EGM regarding micronutrients for depression among children and adolescents with 30 primary research publications. They found that the most frequently studied micronutrients were vitamin D, zinc, iron, folate, and vitamin B‐12. Britton et al. (2021) collected evidence on mindfulness‐based interventions for depression and interpreted its mechanisms with the self‐related processes, including self‐concept, rumination, self‐compassion, self‐efficacy, and self‐esteem. Whether using participant or intervention studies, these two EGMs did not meet the need among adults with depressive symptoms. Therefore, it will be useful to develop an EGM on the treatments for depression among adults.

## 2 OBJECTIVES

The objective of the map is to map available systematic reviews on the effectiveness of treatments for depressive disorders among adults. Specifically, this EGM includes studies on the effectiveness of treatments across a range of outcome domains.

## 3 METHODS

### 3.1 EGM: Definition and purpose

This EGM is an effectiveness map of interventions for depression, in which the primary dimensions are intervention categories (rows) and indicator domains (columns). Secondary dimensions, such as country and target group, are included as filters (White, 2020). We will adapt EGM methods from various key studies (Bragge, 2011; Snilstveit, 2016; White, 2020) and utilize a five‐stage process:

- Define a framework, which determines the scope and inclusion and exclusion criteria;
- Identify available evidence (search).
- Appraise the quality of evidence.
- Extract, code, and summarize the data that relate to the objectives.
- Visualize and present the findings in a user‐friendly format.

We will use the EPPI‐Mapper mapping tool developed by the EPPI‐Centre to display identified studies using the framework described below.

### 3.2 Framework development and scope

After discussions and recommendations with stakeholders and advisory panels on August 8, 2022, a framework for the main classification items of different intervention populations and groups was finalized. We further defined the scope and framework for consultation with our research group, which included researchers from evaluation and evidence synthesis (HW and YKH), education (GLP), public health (LJY), psychiatrist (WW), and psychotherapist (QK and XZ). Finally, the EGM will include two dimensions of the framework: interventions and indicators/outcomes. We will follow the standard EGM framework as a matrix where the rows are intervention domains and the columns are indicator/outcome categories.

### 3.3 Stakeholder engagement

The group members for this EGM include researchers from evaluation and evidence synthesis (Howard White), education (Liping Guo), public health (Jieyun Li), health and wellbeing (Kehu Yang), health statistics and services (Yaogeng Chen), doctors of psychiatric (Wen Wang), and psychotherapist (Kun Qiao and Zheng Xu). Feedback from the group members was received and assimilated into the framework plan at the title registration stage. The stakeholders will be engaged at all stages of the EGM to review and comment on interventions, studies, outputs, map findings and provide advice on dissemination channels.

There has been extensive stakeholder engagement in the preparation of the framework. This has included:

- Stakeholders' online consultation meetings at least bimonthly in discussing the direction and scope of EGM.
- One‐on‐one calls with stakeholders not able to attend the larger stakeholder consultations.
- Three rounds of piloting and revising the coding framework using selected eligible or ineligible studies. The details were shown in Tables 1, 2, 3.
- Further stakeholder consultation is planned for January 2023.

Each of these intended users has participated in defining the intervention and outcome framework. The participation of the advisory group members will contribute to greater alignment of global and national efforts in forming the basis for improving the evidence base on depressive disorder.

### 3.4 Conceptual framework

**Figure 1:**
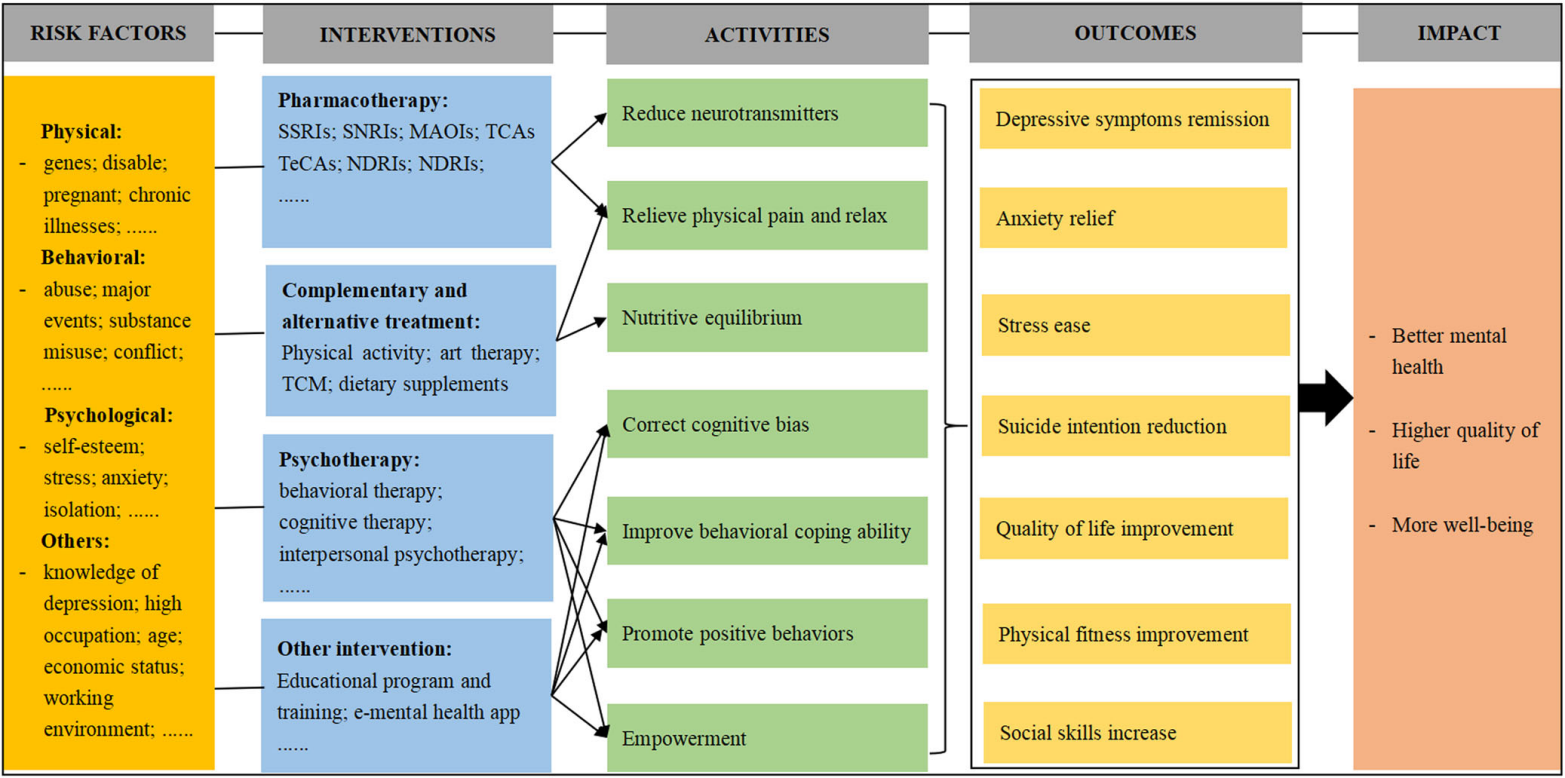
Conceptual framework for depression.

Depression can result from a complex interaction of psychological, physical, behavioral, and other factors (Pandey, 2021; Ratanasiripong, 2012; World Health Organization, 2017), and there are various treatment mechanisms (Britton, 2021; Xie, 2021). For example, pharmacotherapy mainly reduces the activity of brain nerves by reducing neurotransmitters in the body, thus stabilizing the mood of patients with depression; psychological therapy as well as educational program and training aim to correct cognitive bias, improve behavioral coping ability, and promote positive behaviors to empower the suffers of depression. Besides, relieving physical pain, relaxing the body, and balancing nutrition are complementary treatments for depression. The conceptual framework for depression is shown in Figure 1. The present EGM aims to provide a classification of treatments for depression.

### 3.5 Dimensions

#### 3.5.1 EGM framework interventions

This EGM focuses on systematic reviews of effectiveness studies of interventions, with a primary aim of treatment or reducing depressive disorder in adults. The intervention‐outcome framework identified four categories of intervention groups based on the type of interventions. Each category has several subcategories based on the pilot coding of 30 studies, expert advice, and discussion among the team and advisory members.

Table 4 lists the intervention categories, subcategories, and definitions. There are four categories of intervention: psychotherapy, pharmacotherapy, complementary and alternative treatments, and other interventions. The psychotherapy category comprised seven sub‐categories: behavioral therapy (BT), cognitive therapy (CT), cognitive behavioral therapy (CBT), interpersonal psychotherapy (IPT), psychodynamic psycho‐dynamic psychotherapy (PP), supportive psychotherapy (SP), other psychotherapy; the pharmacotherapy covered eight sub‐categories: selective serotonin reuptake inhibitors (SSRIs), serotonin‐norepinephrine reuptake inhibitors (SNRIs), monoamine oxidase inhibitors (MAOIs), tricyclic antidepressants (TCAs), tetracyclic antidepressants (TeCAs), norepinephrine and dopamine reuptake inhibitors (NDRIs), serotonin antagonist and reuptake inhibitors (SARIs), and other antidepressants; the complementary and alternative treatments include five sub‐categories: physical therapy (PT), physical activity (PA), art therapy (AT), traditional Chinese medicine (TCM), and dietary supplement; and the other intervention comprised educational program and training, e‐mental health apps and other treatments.

**Table 1:** Studies used during piloting: Characteristics of included studies.

Appleton et al. (2016)	
Participants	General adult
Interventions	Complementary and alternative treatments
	‐Dietary supplement (*ω*−3 fatty acids)
Comparisons	Placebo
Outcomes	Remission: depressive symptomology (SMD = −0.32); Adverse events: OR = 1.24
Type of depression	Major depressive disorder
Country	Netherlands, USA, Brazil, Iran, Australia, Canada, Korea, UK, Italy, New Zealand, China
Quality of studies	Low quality
Conflict of interest	No
Funding	Yes [The National Institute for Health Research (NIHR)]
Chu et al. (2017)	
Participants	Infertile adult with ART treatment
Interventions	Complementary and alternative treatments
	‐Traditional Chinese medicine (Acupuncture)
	Psychological therapy
	Other intervention (lifestyle intervention)
Comparisons	Unclear
Outcomes	Remission: depression (SMD = 0.09);
	Symptoms of depressive disorder: anxiety
	‐Complementary and alternative treatments (Physical therapy): SMD = 0.48;
	‐Psychological therapy: SMD = 0.25;
	‐Other intervention (lifestyle intervention): (SMD = −0.81).
Type of depression	Unspecified
Country	Denmark, Turkey, Brazil, Netherlands, China, UK, South Africa, Germany, USA, Australia, Iran, Greece, Italy
Quality of studies	High quality
Conflict of interest	No
Funding	Unclear
Cuijpers (2013)	
Participants	Older adults, Student population, Women with PPD, General medical population
Interventions	Psychotherapy (Cognitive‐Behavioral Therapy), pharmacotherapy, Combination therapy (CBT plus pharmacotherapy)
Comparisons	Wait‐list, TAU, Other control group, no comparison
Outcomes	Remission: depression
	‐CBT versus Control Condition: *g* = 0.71;
	‐Pharmacotherapy versus combined: *g* = 0.49);
	‐CBT versus pharmacotherapy: *g* = 0.03.
Type of depression	Unspecified
Country	USA, UK, Canada, Australia
Quality of studies	High quality
Conflict of interest	Unclear
Funding	The Canadian Psychiatric Association supports
Ekers (2008)	
Participants	General adult
Interventions	Psychotherapy (Behavioral therapy, BT)
Comparisons	TAU, psychotherapy (CBT/CT, Brief psychotherapy), other comparison (Supportive counseling)
Outcomes	Remission: depression
	‐BT versus CAU: SMD = −0.70;
	‐BT versus Brief psychotherapy: SMD = −0.56;
	‐BT versus Supportive counseling: SMD = −0.75;
	‐BT versus CBT: SMD = 0.08.
Type of depression	Unspecified
Country	Not mentioned
Quality of studies	Low quality
Conflict of interest	No
Funding	Unclear
Hu (2019)	
Participants	Adult cancer patients
Interventions	Other intervention (Caregiver–patient dyad intervention)
Comparisons	TAU, wait list, active control
Outcomes	Remission: depression (SMD = − 0.35);
	Symptoms of depressive disorder: anxiety (SMD = −0.42); Life and Social skills: QOL (SMD = 0.25); relatedness (SMD = 0.18)
Type of depression	Unspecified
Country	Not mentioned
Quality of studies	Critically Low quality
Conflict of interest	No
Funding	Unclear
Kho (2003)	
Participants	depressed patients (mean age > 35)
Interventions	Complementary and alternative treatments
	‐Physical therapy (electroconvulsive therapy, ECT)
Comparisons	Complementary and alternative treatments (Physical therapy, Physical activity), psychotherapy (CBT), pharmacotherapy (Imipramine),
Outcomes	Remission: depression
	‐ECT versus control group: SMD = 0.90;
	‐ECT versus Sine wave: SMD = 0.81;
	‐ECT versus Brief pulse: SMD = 1.08;
	‐ECT versus pharmacotherapy: SMD = 0.68;
	‐ECT versus Sim. ECT: SMD = 0.95.
Type of depression	Unspecified
Country	Not mentioned
Quality of studies	Critically Low quality
Conflict of interest	Unclear
Funding	Unclear
Kishi (2017)	
Participants	General adult (mean age = 39.9)
Interventions	Pharmacotherapy (Memantine)
Comparisons	Placebo
Outcomes	Remission: depression response rate
	‐Major depressive disorder: RR = 0.94;
	‐Bipolar disorder: RR = 0.83.
Type of depression	Major depressive disorder, Bipolar disorder
Country	Iran, USA, China
Quality of studies	Critically Low quality
Conflict of interest	Unclear
Funding	Unclear

**Table 2:** Continue: Studies used during piloting: Characteristics of included studies.

Mukai (2014)	
Participants	General adult (depressed patients: mean age > 18)
Interventions	Complementary and alternative treatments
	‐Dietary supplement (inositol)
	Pharmacotherapy
	‐Selective serotonin reuptake inhibitor (SSRI)
Comparisons	Placebo
Outcomes	Remission: premenstrual dysphoric disorder (SMD = −1.15); bipolar disorder (SMD = 0.11); major depressive disorder (SMD = 0.17).
Type of depression	Bipolar disorder (BD), major depressive disorder (MDD), premenstrual dysphoric disorder (PMDD)
Country	Israel, USA
Quality of studies	Critically Low quality
Conflict of interest	No
Funding	No
Ng (2018)	
Participants	General adult (mean age > 19 years old)
Interventions	Complementary and alternative treatments
	‐Dietary supplement (Probiotics)
Comparisons	Placebo
Outcomes	Remission: depression (SMD = −0.68)
Type of depression	Mild to moderate depressive symptoms
Country	Iran, UK, Korea, France, Japan, Sweden, Canada, New Zealand, Netherlands
Quality of studies	High quality
Conflict of interest	No
Funding	Unclear
Pae (2014)	
Participants	General adult (mean age > 35 years old)
Interventions	Pharmacotherapy (Aripiprazole augmentation, AA)
Comparisons	Pharmacotherapy (Aripiprazole)
Outcomes	Remission: major depressive disorder
	‐Open‐label studies (SMD = −2.114);
	‐RCTs (SMD = −2.202).
Type of depression	Major depressive disorder
Country	Not mentioned
Quality of studies	Critically Low quality
Conflict of interest	Unclear
Funding	Yes [the Korean Health Technology R&D Project, Ministry of Health & Welfare, Republic of Korea (HI12C0003)]

**Table 3:** Studies used during piloting: Characteristics of excluded studies.

Study	Reason for exclusion
Barry (2019)	Wrong study design
Greenberg (1994)	Wrong study design
McNaughton (2009)	Wrong study design
Wu (2012)	Wrong study design
Beutler (2018)	Wrong population
Yýldýz (2001)	Wrong outcome
Gellersen (2018)	Wrong population
Zabalegui (2008)	Non‐English
Ghosh (2007)	Wrong study design
Perlman (2019)	Wrong study design
Lauche (2013)	Wrong population
Lauche (2013)	Wrong population
Churchill (2002)	Wrong intervention
Tahan (2020)	Wrong population
Gloaguen (1998)	Wrong population
Kappelmann (2018)	Wrong population
Luo (2020)	Wrong population
Necho (2020)	Wrong population
Chen (1999)	Wrong outcome
Li (2014)	Wrong outcome

**Table 4:** Intervention categories and sub‐categories.

Categories	Sub‐category	Definitions	Example from included study
Psychotherapy	Behavioral therapy (BT)	Behavior therapy (BT) is a form of psychotherapy that applies the principles of learning, operant conditioning, and classical conditioning to eliminate symptoms and modify ineffective or maladaptive patterns of behavior (American Psychological Association, 2015).	(Ekers, 2008) “This systematic review sought to identify all randomized trials of behavioral therapy for depression, determine the effect of such interventions and examine any moderators of such effect.”
	Cognitive therapy (CT)	Cognitive therapy (CT) a form of psychotherapy based on the concept that emotional and behavioral problems in an individual are, at least in part, the result of maladaptive or faulty ways of thinking and distorted attitudes toward oneself and others (American Psychological Association, 2015).	(Gloaguen, 1998) “To be included in the study, trials had to be randomized and have at least one CT group, and one comparison group: waiting list or placebo, anti‐depressants, behavior therapy or another psycho‐therapeutic treatment.”
	Cognitive‐behavioral therapy (CBT)	Cognitive behavior therapy (CBT) is a form of psychotherapy that integrates theories of cognition and learning with treatment techniques derived from cognitive therapy and behavior therapy (American Psychological Association, 2015).	(Yu, 2020) “Our study aims to evaluate the clinical efficacy and quality of life of cognitive‐behavioral therapy (CBT) for patients who have acute coronary syndrome (ACS) with anxiety and depression.”
	Interpersonal psychotherapy (IPT)	Interpersonal psychotherapy (IPT) is a time‐limited form of psychotherapy, originally based on the interpersonal theory of Harry Stack Sullivan, positing that relations with others constitute the primary force motivating human behavior (American Psychological Association, 2015).	(van Hees, 2013) “This systematic review describes a comparison between several standard treatments for major depressive disorder (MDD) in adult outpatients, with a focus on interpersonal psychotherapy (IPT).”
	Psycho‐dynamic psychotherapy (PP)	Psycho‐dynamic psychotherapy (PP) is the form of psychotherapy, falling within or deriving from the psychoanalytic tradition, that view individuals as reacting to unconscious forces (e.g., motivation, drive), that focus on processes of change and development, and that place a premium on self‐understanding and making meaning of what is unconscious (American Psychological Association, 2015).	(Driessen, 2015) “The efficacy of short‐term psycho‐dynamic psychotherapy (STPP) for depression is debated. Recently, a number of large‐scale and high‐quality studies have been conducted. We examined the efficacy of STPP by updating our 2010 meta‐analysis.”
	Supportive psychotherapy (SP)	Supportive psychotherapy (SP) is a form of therapy that aims to relieve emotional distress and symptoms without probing into the sources of conflicts or attempting to alter basic personality structure (American Psychological Association, 2015).	(Cuijpers, 2008) “7 major types of psychological treatment for mild to moderate adult depression (cognitive‐behavior therapy, non‐directive supportive treatment, behavioral activation treatment, psycho‐dynamic treatment, problem‐solving therapy, interpersonal psychotherapy, and social skills training) were directly compared with other psychological treatments.”
	Other psychotherapy	/	/
Pharmacotherapy	Selective serotonin reuptake inhibitors (SSRIs)	Selective serotonin reuptake inhibitors (SSRI) are any of a class of antidepressants that are thought to act by blocking the retake of serotonin into serotonin‐containing pre‐synaptic neurons in the central nervous system (American Psychological Association, 2015).	(Omori, 2009) “We conducted a systematic review to synthesize the best available evidence on the efficacy of fluvoxamine for adult patients suffering from major depression in comparison with other active anti‐depressive agents.”
	Serotonin‐norepinephrine reuptake inhibitors (SNRIs)	Serotonin‐nor epinephrine reuptake inhibitors (SNRIs) are a class of antidepressants that exert their therapeutic effects by interfering with the presynaptic of both serotonin and norepinephrine by the neurons that released them (American Psychological Association, 2015).	(Huang, 2014) “This meta‐analysis comprehensively shows the efficacy, acceptability, and safety of agomelatine in comparison with SSRIs and SNRIs used as antidepressants in MDD.”
	Monoamine oxidase inhibitors (MAOIs)	Monoamine oxidase inhibitors (MAOIs) is a group of antidepressant drugs that function by inhibiting the activity of the enzyme monoamine oxidase in presynaptic neurons, thereby increasing the amounts of monoamine neurotransmitters (serotonin, norepineph rine, and dopamine) available for release at the presynaptic terminal (American Psychological Association, 2015).	(Heijnen, 2015) “For this systematic review comparing tranylcypromine with placebo or active comparators in bipolar depression, relevant randomized controlled trials were identified from systematic searches of PubMed, EMBASE, and Cochrane library databases.”
	Tricyclic antidepressants (TCAs)	Tricyclic antidepressant (TCA) is any of a group of drugs are the original first‐line medications for treatment of depression. They act by blocking the reuptake of monoamine neurotransmitters (serotonin, dopamine, and norepinephrine) into the presynaptic neuron, thereby increasing the amount of neurotransmitter available for binding to postsynaptic receptors (American Psychological Association, 2015).	(Furukawa, 2002) “To compare the effects and side effects of low dosage tricyclic antidepressants with placebo and with standard dosage tricyclics in acute phase treatment of depression.”
	Tetracyclic antidepressants (TeCAs)	Tetracyclic antidepressants (TeCAs) are a class of antidepressants closely related to TCAs, and the mechanisms of action of these drugs are similar to TCAs with monoamine modulation as their hallmark feature.	(Macedo, 2011) “To evaluate the efficacy and frequency of adverse events with pirlindole in comparison with active comparators.”
	Norepinephrine and dopamine reuptake inhibitors (NDRIs)	Norepinephrine and dopamine reuptake inhibitors (NDRIs) are drugs that function by inhibiting the reuptake of the neuro‐transmitters norepinephrine and dopamine. This leads to increased neural concentrations of these activating neurotransmitters, resulting in increased stimulation of the central nervous system.	(Smith, 2021) “The objective of this systematic review is to assess the efficacy and safety of methylphenidate (MPH) in the treatment of geriatric depression.”
	Serotonin antagonist and reuptake inhibitors (SARIs)	Serotonin antagonist and reuptake inhibitors (SARIs) are a class of drugs used mainly as antidepressants, but also as anxiolytics and hypnotics. They act by antagonizing serotonin receptors such as 5‐HT2A and inhibiting the reuptake of serotonin, norepinephrine, and/or dopamine.	(Sobieraj, 2019) “The interventions include selective serotonin reuptake inhibitors (SSRIs), serotonin norepinephrine reuptake inhibitors (SNRIs), bupropion, mirtazapine, trazodone, vilazodone, or vortioxetine compared with another antidepressant, placebo, or nonpharmacologic therapy.”
	Other antidepressants	/	/
Complementary and alternative treatments	Physical therapy (PT)	physical therapy (PT) is the treatment of pain, injury, or disease using physical or mechanical methods, such as exercise, heat, water, massage, or electric current (American Psychological Association, 2015).	(Tu, 2021) “This systematic review and meta‐analysis aimed to investigate the efficacy of acupuncture on CSAP‐associated anxiety and depression.”
	Physical activity (PA)	Physical activity is defined as any bodily movement produced by contraction of skeletal muscle that increases energy expenditure above the basal level (American Psychological Association, 2015).	(Schuch, 2016) “To evaluate the antidepressant effects of exercise in older adults, using randomized controlled trial (RCT) data.”
	Art therapy (AT)	Art therapy is the use of artistic activities, such as painting and clay modeling, in psychotherapy and rehabilitation (American Psychological Association, 2015).	(Chan, 2011) “The objective of this review was to determine the effectiveness of music listening in reducing depressive symptoms in adults.”
	Traditional Chinese Medicine (TCM)	Traditional Chinese medicine (TCM) is a system of medicine at least 23 centuries old that aims to prevent or heal disease by maintaining or restoring yinyang balance.	(Armour, 2019) “This systematic review and meta‐analysis examined the effectiveness of acupuncture in major depressive disorder.”
	Dietary supplement	Dietary supplements are chemical substances like minerals, vitamins, and antioxidants, which are part of normal nutrition but also can added to normal nutrition in the shape of more or less pure substances (Hoffmann, 2019).	(Appleton, 2016) “The objective of this review is to assess the effects of n‐3PUFAs compared with comparator (eg, placebo, antidepressant treatment, standard care) for MDD in adults.”
Other intervention	Educational program and training	Education program and training is the process of teaching or acquiring knowledge and skills related to depression disorder (American Psychological Association, 2015).	(Barry, 2019) “The present meta‐analysis and systematic review provides the first synthesis of all existing studies in which Memory Specificity Training has been tested within the context of emotional disorders.”
	e‐Mental health apps	e‐Mental health apps are smartphone apps aim to improve quality and increase access to mental health care (Bakker, 2016).	(Linardon, 2019) “We conducted a meta‐analysis of 66 randomized controlled trials of app‐supported smartphone interventions for mental health problems.”
	Other treatments	/	/

#### 3.5.2 EGM framework indicators/outcomes

For inclusion, the primary aim of the systematic review is to assess the effects of interventions on depressive symptoms in adults as a primary indicator/outcome. The indicator/outcome domains and sub‐domains are the main categories and subcategories, used as column headings in our map.

The most common outcomes are remission of depressive symptoms (i.e., dysregulation disorder, major depressive disorder, persistent depressive disorder, premenstrual dysphoric disorder, a depressive disorder with another medical condition, and bipolar depression), symptoms of depressive disorder (anxiety, stress, suicide intention, and sleep disturbance), life and social skills (quality of life, physical function, and social function), and adverse events of pharmacotherapy and other treatments. See Table 5 for the list of outcomes, definitions, and examples.

**Table 5:** Indicator/outcome domain.

Indicator/outcome domain	Sub‐domain	Definitions	Example from included study
Adherence to treatment		Adherence to treatment is the extent to which a person's behavior—taking medication, following a diet and/or executing lifestyle changes, corresponds with agreed recommendations from a healthcare provider (Ahmed, 2014)	/
Remission of depressive symptom	Major depressive disorder	Major depressive disorder (MDD), also known simply as depression, is a mental disorder, characterized by at least two weeks of pervasive low mood, low self‐esteem, and loss of interest or pleasure in normally enjoyable activities (American Psychological Association, 2015).	(Appleton, 2016) “To assess the effects of w‐3 polyunsaturated fatty acids compared with a comparator for major depressive disorder (MDD) in adults.”
	Persistent depressive disorder	Dysthymia, also known as persistent depressive disorder (PDD), is a mental and behavioral disorder, specifically a disorder primarily of mood, consisting of the same cognitive and physical problems as depression, but with longer‐lasting symptoms (American Psychological Association, 2015).	/
	Premenstrual dysphoric disorder	Premenstrual dysphoric disorder (PMDD) is a severe and disabling form of premenstrual syndrome. Bipolar depression, anxiety disorders, and other Axis I disorders are more common in those with PMDD (American Psychological Association, 2015).	/
	Depressive disorder with another medical condition	Depressive disorder with another medical condition, such as cancer, diabetes, pregnancy (American Psychological Association, 2015).	(Wang, 2016) “This study aims at concluding the current evidence on the therapeutic effects of acupoints stimulation for cancer patients with anxiety and depression.”
	Bipolar depression	Bipolar disorder, previously known as manic depression, is a mood disorder characterized by periods of depression and periods of abnormally‐elevated mood that last from days to weeks each (American Psychological Association, 2015).	(Kishimoto, 2016) “Parallel‐group or crossover randomized controlled trials comparing single intravenous infusion of ketamine or a non‐ketamine NMDAR antagonist *v*. placebo/pseudo‐placebo in patients with major depressive disorder (MDD) and/or bipolar depression (BD) were included in the analyses.”
	Other specific depressive symptoms	/	/
	Unspecified	Unspecified depressive symptom.	(Ekers, 2008) “Studies included participants who were adults, treated in community or in‐patient settings with a primary diagnosis of depression.”
Symptoms of depresson	Anxiety	Anxiety is an emotion characterized by an unpleasant state of inner turmoil and includes subjectively unpleasant feelings of dread over anticipated events (American Psychological Association, 2015).	(Zhao, 2019) “This meta‐analysis revealed that laughter and humor interventions are effective in relieving depression, anxiety, and improve sleep quality in adults.”
	Stress	Stress is a feeling of emotional strain and pressure (American Psychological Association, 2015).	(Zhang, 2020) “The present meta‐analysis suggested that iMBIs had small to moderate effects in reducing stress and improving mindfulness of the general population in comparison with the control group.”
	Other mental distress	Mental distress is a term used, by some mental health practitioners and users of mental health services, to describe a range of symptoms and experiences of a person's internal life that are commonly held to be troubling, confusing, or out of the ordinary (American Psychological Association, 2015).	(Hu, 2019) “Meta‐analysis showed that interventions were not effective at reducing patient hopelessness.”
	Suicide intention	Suicide intention is the thought or act of intentionally causing one's own death (American Psychological Association, 2015).	/
	Sleep disturbance	Sleep disturbance, or sleep disorder, is a medical disorder of an individual's sleep patterns (American Psychological Association, 2015).	(Zhao, 2019) “This meta‐analysis revealed that laughter and humor interventions are effective in relieving depression, anxiety, and improve sleep quality in adults.”
Life and social skills	Quality of life	Quality of life (QOL) is an individual's perception of their position in life in the context of the culture and value systems in which they live and in relation to their goals, expectations, standards, and concerns (American Psychological Association, 2015).	(Hu, 2019) “dyadic intervention was associated with statistically and clinically significant improvements in patient total QOL.”
	Physical fitness	Physical fitness is a state of health and well‐being and, more specifically, the ability to perform aspects of sports, occupations, and daily activities (American Psychological Association, 2015).	(Hu, 2019) “Only four trials reported changes in pain level post‐intervention and analysis showed that caregiver‐patient dyad care was not associated with a change in pain.”
	Social skill	Social skill is any competence facilitating interaction and communication with others where social rules and relations are created, communicated, and changed in verbal and nonverbal ways(American Psychological Association, 2015).	(Hu, 2019) “Eight studies assessed patients' relatedness with their important persons during the first 3 months following the intervention, the effect size was small but significant.”
Adverse events		Adverse event (AE) is any untoward medical occurrence in a patient or clinical investigation subject administered a pharmaceutical product and which does not necessarily have a causal relationship with this treatment (American Psychological Association, 2015).	(Appleton, 2016) “Numbers of individuals experiencing adverse events were similar in intervention and placebo groups.”

#### 3.5.3 EGM framework population dimension

The primary population of interest for this map is adults (over 18 years old). We will sub‐categorize the adult population according to age group, gender and sexual orientation, career, and health state (as shown in Table 6). The population dimension will be listed as a filter. For studies assessing effects with people who are providing services, we will sub‐categorize according to whether they are professionals or volunteers.

**Table 6:** Population dimension.

Population categories	Definitions
Age	Adult age refers to people aged 18 and over. The following sub‐groups will be coded: 18–60 years and ≥60 years
Gender	Male and female based on biological indicators
LGBT community	Studies where the principal population group being studied identify as LGBT
Career	Studies where the principal population group being studied identify as specified career
Health state	Studies where the principle population group being studied are people with physical disease and/or other psychical disorder

### 3.6 Inclusion and exclusion criteria

#### 3.6.1 Types of study design

We will include only systematic reviews that conduct meta‐analyses of randomized controlled trials (RCTs). According to the definition of systematic review from Campbell Collaboration and our objective, a systematic review met the following four criteria will be included: (1) clear inclusion and exclusion criteria; (2) an explicit search strategy; (3) systematic coding and analysis of included studies; (4) meta‐analysis. If a systematic review included the primary studies using RCT and quasi‐experimental designs (QED), we will only include the data from RCTs.

Protocols for ongoing research will be included. Qualitative reviews, integrative reviews, rapid reviews, reviews of reviews, and evidence synthesis/summaries are beyond the inclusion criteria. Primary studies are excluded.

#### 3.6.2 Types of intervention/problem

We will include systematic reviews assessing any interventions with an explicit aim of reducing depressive symptoms, including educational programs and training, psychotherapy, physical therapy, pharmacological treatment, physical activity, and any interventions that aim to treat depression in adults.

#### 3.6.3 Types of the population

The target population is adults (aged 18 and over) with depressive symptoms including those with any other mental and/or physical problems, and professionals or volunteers providing services to the target population. If a review covered both adults and children, we will extract the independent information on adults if available.

#### 3.6.4 Types of outcome measures

We will include systematic reviews that assess the effect of interventions on depressive symptoms and related symptoms, such as anxiety, stress, suicide intention, life and social skills, and adverse events by the pharmacy or other treatments.

#### 3.6.5 Other eligibility criteria

Studies will not be limited by geographic location. Due to the limitation of team members, only studies published in English and Chinese will be included. There is no restriction on publication time.

##### Types of location/situation (as applicable)

Not applicable.

##### Types of settings

Any settings, such as hospitals, communities, and institutions, will be included.

### 3.7 Search methods and sources

The search for EGM will be conducted in three stages:

- Stage 1: Pilot for screening and coding of included studies (deadline: January 2023).
- Stage 2: Search for relevant systematic reviews from academic databases and international organizations (deadline: February 2023).
- Stage 3: Search for additional websites for gray literature after expert consultation (deadline: March 2023).

#### 3.7.1 Database search for stage 2

The following international electric databases will be searched:

- Social Sciences Citation Index (via Web of Science)
- ScienceDirect (https://www.sciencedirect.com/)
- Taylor & Francis Online Database (https://www.tandfonline.com/)
- JSTOR (https://www.jstor.org/)
- PsychArticles (via ProQuest)
- PsychInfo (via EBSCOhost)
- OCLC FirstSearch (https://firstsearch.oclc.org/)
- PubMed (https://pubmed.ncbi.nlm.nih.gov/)
- EMBASE (https://www.embase.com/)
- The Cochrane Library (https://www.cochranelibrary.com/)
- The Campbell Library (https://www.campbellcollaboration.org/better-evidence.html)
- 3ie Systematic Review Database (https://www.3ieimpact.org/evidence-hub/publications/systematic-reviews)
- Epistemonikos (https://www.epistemonikos.org/)
- CNKI (https://www.cnki.net/)

In addition, the pre‐print repository MedRxiv (https://www.medrxiv.org/) will also be searched

Below, the search strategy for PubMed is provided:

***Depressive symptom keywords***

‐ “Depressive Disorder”[Mesh] OR “Long‐Term Synaptic Depression”[Mesh] OR “Depression, Postpartum”[Mesh] OR “Depressive Disorder, Treatment‐Resistant” [Mesh] OR “Bipolar Disorder”[Mesh] OR “Dysthymic Disorder”[Mesh] OR “Seasonal Affective Disorder”[Mesh] OR “Depressive Disorder, Major”[Mesh] OR “Adjustment Disorders”[Mesh] OR “Affective Disorders, Psychotic”[Mesh] OR “Depression”[Mesh]
‐ OR
‐ (((depress* [Title/Abstract] OR distress [Title/Abstract]) OR (“Psychological Distress”[Mesh])) OR (“Depressive Disorder”[Mesh] OR “Long‐Term Synaptic Depression”[Mesh] OR “Depression, Postpartum”[Mesh] OR “Depressive Disorder, Treatment‐Resistant”[Mesh] OR “Bipolar Disorder”[Mesh] OR “Dysthymic Disorder”[Mesh] OR “Seasonal Affective Disorder”[Mesh] OR “Depressive Disorder, Major”[Mesh] OR “Adjustment Disorders”[Mesh] OR “Affective Disorders, Psychotic”[Mesh] OR “Depression”[Mesh])

***Population keywords***

‐ (“Adult” [Mesh] or (adult*[Title/Abstract]) or (old [Title/Abstract])
‐ NOT
‐ (“Child “ [Mesh] or (Child*[Title/Abstract]))
‐ NOT
‐ (“Adolescent “ [Mesh] or Adolescen* [Title/Abstract] OR Teen*[Title/Abstract] OR Youth [Title/Abstract] OR underage [Title/Abstract])

***Study design keywords***

‐ ((“systematic review”[Title] OR “meta‐analysis” [Title] OR meta [Title] OR systematic [Title]) OR (“Meta‐Analysis” [Publication Type] OR “Meta‐Analysis as Topic”[Mesh])) OR (“Systematic Review” [Publication Type] OR “Systematic Reviews as Topic”[Mesh])

The following Chinese electric databases will be searched:

- Chinese National Knowledge Infrastructure, CNKI (https://new.wanfangdata.com.cn/index.html)
- Database for Chinese Technical Periodicals, VIP (http://www.tydata.com/)
- Wanfang (https://new.wanfangdata.com.cn/index.html)

Below, the search strategy for CNKI is provided:

***Population keywords**:*

‐ 篇关摘= 成人 + 成年人 + 大学生
‐ Not
‐ 篇关摘= 儿童 + 青少年 + 未成年 + 少年

***Depressive symptom keywords**:*

‐ 篇关摘= 抑郁 + 心理

***Study design keywords**:*

‐ 篇关摘= 系统评价 + 元分析 + 荟萃分析 + meta分析

#### 3.7.2 Searching other resources

We will consult the following sources of grey literature and search websites of organizations for mental health research to identify relevant unpublished studies and reports. The following grey literature resources will be searched using the keyword “depression”:

- World Health Organization (https://www.who.int/)
- American Psychological Association (https://www.apa.org/)
- Centers for Disease Control and Prevention (https://www.cdc.gov/)
- National Institute of Mental Health (https://www.nimh.nih.gov/)
- Open Grey (http://www.opengrey.eu/)

In addition, we will also survey Google Scholar using the keyword “depression intervention” and scan the first 50 pages for relevant studies. Moreover, relevant reviews cited in previous systematic reviews on depression will be scanned, and reference lists of included studies will also be searched.

### 3.8 Analysis and presentation

#### 3.8.1 Unit of analyses

Each entry in the map will be a systematic review of effectiveness. If a review contains multiple interventions, the reviewers will extract all data as dependent reports that are eligible for this EGM. The final EGM will identify the number of studies covered by the map in each sector or subsector.

#### 3.8.2 Report structure

The EGM report will include the following sections: executive summary, background, intervention, results, and conclusion. The executive summary will summarize the report, providing key findings and implications for future policy planning and research. The background of this study will comprehensively describe the current situation of depressive disorder in adults and its impact on society. Examples of existing interventions are described and the goals of EGM are clarified. In addition, we will describe the scope by defining an intervention and outcome framework.

The description of the methods will include a definition of the data sources and methods of searching, the inclusion and exclusion criteria, study selection, study confidence appraisal, data extraction methods, and the approach to presentation/visualization. This section will provide a table in the text showing one full search from a database and a PRISMA flow chart. An appendix will provide full search strategies used for each database, including any restrictions and filters used.

The outcome will show the number, type, and quality of the studies retrieved for the outcome categories, the population targeted for the specific study, and the change in outcomes after the intervention.

The conclusion is expected to provide insights for researchers and decision‐makers, build on the evidence base in this field, and identify key areas for future research. Moreover, we will include studies considering the impact of conducting EGM.

The following tables and figures will be included:

- Figure: PRISMA flow chart table.
- Table: Number of studies by intervention and subcategories.
- Table: Number of studies by population.
- Table: Number of studies by intervention category and study confidence.

#### 3.8.3 Filters for presentation

We will present results as a matrix of interventions (rows) and outcomes (columns) and assess the availability of evidence across the additional filters. In addition to the interventions and outcomes, the following filters will be coded (details are in Tables 7 and 8):

- Population subgroups of interest include age group (young and middle‐aged adults: 18–60 years; older adults: 60 years and above), gender and sexual orientation (female, male, and LGBT community), career (e.g., nurse, doctor, student, teacher, police), and health state (depression alone, depression with physical disease, and depression with other psychical disorders).
- The number of interventions: single treatment and combination treatment.
- Type of depression: disruptive mood dysregulation disorder, major depressive disorder, persistent depressive disorder (dysthymia), premenstrual dysphoric disorder, substance or medication‐included depressive disorder, depressive disorder due to another medical condition, bipolar depression, other specific depressive symptoms, unspecified depressive disorder.
- The severity of depression: mild depressive symptoms, moderate depressive symptoms, moderate to severe depressive symptoms, severe depressive symptoms, major depressive symptoms, other specific depressive symptoms, and undefined.
- Period of depression: lifetime, 12 months, 6 months, 1 month, and not stated.
- Number of episodes: depression episode, depression recurrence, and not stated;
- The implementer of treatment: self‐help, healthcare provided, and provided by mental health professionals or volunteers.
- Effectiveness of interventions: We will record whether the systematic review reported a mean positive statistically significant effect, a mean statistically significant negative effect, or no statistically significant difference between treatment and comparison conditions.
- Region: Africa, Americas, East Asia, Europe, Eastern Mediterranean, Western Pacific.
- Countries: any noted.
- Conflict of interest: yes, no, and unclear.
- Quality of studies: high, moderate, low, and critically low.

**Table 7:** List of filters.

List of type of populations	
**Categories**	**Sub‐category**
Age	young and middle‐aged adults (18‐65 years old)
	older adults (over 65 years old)
Gender and sexual orientation	female
	male
	LGBT community
Career	any noted
Health state	depression alone
	depression with physical disease
	depression with other psychical disorder

**List of the effectiveness of interventions**	
**Categories**	**Sub‐category/comparation group**
Positive	Placebo
	Psychotherapy
	Physical therapy
	Physical activity
	Pharmacotherapy
	Waitlist
	Treatment as usual (TAU)
	Other comparison
	No comparison
Negative	Placebo
	Psychotherapy
	Physical therapy
	Physical activity
	Pharmacotherapy
	Waitlist
	Treatment as usual (TAU)
	Other comparison
	No comparison
No significant difference	Placebo
	Psychotherapy
	Physical therapy
	Physical activity
	Pharmacotherapy
	Waitlist
	Treatment as usual (TAU)
	Other comparison
	No comparison

**Table 8:** Continue: List of filters.

List of other filters	
**Others**	**Categories**
Number of episodes	Depression episode
	Depression recurrence
	Not applicable
	Not state
Severity of depression	Mild depressive disorder
	Moderate depressive disorder
	Moderate to severe depressive disorder
	Severe depressive disorder
	Major depressive disorder
	Undefined
Duration of depression	Lifetime
	24 months
	12 months
	6 months
	Unspecified
Region	Africa
	Americas
	East Asia
	Europe
	Eastern Mediterranean
	Western Pacific
Country	Any country noted
Quality of studies	Critical low
	Low quality
	Middle quality
	High quality
Conflict of interest	Yes
	No
	Unclear
Funding	Yes
	No
	Unclear

#### 3.8.4 Dependency

Each entry in the map will be a systematic review of effectiveness. The final EGM will identify the number of studies covered by the map in each sector or subsector.

### 3.9 Data collection and analysis

#### 3.9.1 Screening and study selection

Two reviewers (Ren JJ and Huang XY) will independently screen the titles and abstracts of all retrieved articles. Furthermore, titles and abstracts will be screened by Rayyan based on population, intervention, and study design but not the outcome (Ouzzani, 2016). Full texts of potentially eligible studies will then be retrieved and screened in Excel. The reviewers will compare the results, and conflicts will be resolved through discussion or by a third reviewer (Xu Z). Authors of studies or reviews are not contacted for missing information.

#### 3.9.2 Data extraction and management

Coding is the process of capturing the required data from included studies, such as study population, intervention, and study design. Coding will be done independently by two coders (Li JY and Xu Z) using EPPI Mapper, with a third‐party arbitrator (Guo LP) in the event of disagreement. The studies will be coded based on intervention category and sub‐category, indicator/outcome domain, and sub‐domain; treatment population, country, and population characteristics. In addition, coding includes critical appraisal, which is described in Section 3.5. Guo LP will be responsible for data management.

#### 3.9.3 Tools for assessing the risk of bias/study quality of included reviews

The reliability of the study findings in the included systematic reviews will be assessed using a measurement tool to assess systematic reviews (AMSTAR‐2). This progress will be conducted independently by two reviewers (Li JY and Xu Z), with any disagreements will be resolved by a third reviewer (Yang KH). The items of AMSTAR‐2 are shown in Table 9 (Shea, 2017).

**Table 9:** Items of AMSTAR‐2.

No.	Item	Evaluation
1	Did the research questions and inclusion criteria for the review include the components of PICO?	Yes/no
2	Did the report of the review contain an explicit statement that the review methods were established before the conduct of the review and did the report justify any significant deviations from the protocol?	Yes/partial/no
3	Did the review authors explain their selection of the study designs for inclusion in the review?	Yes/no
4	Did the review authors use a comprehensive literature search strategy?	Yes/partial/no
5	Did the review authors perform study selection in duplicate?	Yes/no
6	Did the review authors perform data extraction in duplicate?	Yes/no
7	Did the review authors provide a list of excluded studies and justify the exclusions?	Yes/no
8	Did the review authors describe the included studies in adequate detail?	Yes/partial/no
9	Did the review authors use a satisfactory technique for assessing the risk of bias (ROB) in individual studies that were included in the review?	Yes/partial/no/includes only NRSI or RCT
10	Did the review authors report on the sources of funding for the studies included in the review?	Yes/no
11	If meta‐analysis was performed did the review authors use appropriate methods for statistical combination of results?	Yes/no/no meta‐analysis conducted
12	If meta‐analysis was performed, did the review authors assess the potential impact of ROB in individual studies on the results of the meta‐analysis or other evidence synthesis?	Yes/no/no meta‐analysis conducted
13	Did the review authors account for ROB in individual studies when interpreting/discussing the results of the review?	Yes/no
14	Did the review authors provide a satisfactory explanation for, and discussion of, any heterogeneity observed in the results of the review?	Yes/no
15	If they performed quantitative synthesis did the review authors carry out an adequate investigation of publication bias (small study bias) and discuss its likely impact on the results of the review?	Yes/no/no meta‐analysis conducted
16	Did the review authors report any potential sources of conflict of interest, including any funding they received for conducting the review?	Yes/no

#### 3.9.4 Methods for mapping

We will use the EPPI Mapper mapping tool developed by the EPPI‐Centre to display identified studies using the framework described above.

## CONTRIBUTIONS OF AUTHORS

***Content expertise***

- Zheng Xu is a teacher at the Psychological Counselling Centre of Lanzhou University.
- Kun Qiao is the deputy director of the Institute of Medical Physiology and Psychology at the School of Basic Medical Sciences of Lanzhou University.
- Wen Wang is an associate doctor of the psychiatry department of the First Hospital of Lanzhou University.
- Yaogeng Chen is a professor focused on the mental crisis at the School of Science at Ningxia Medical University.

***Systematic review expertise***

All authors are experienced systematic reviewers, which means they are proficient in carrying out the various processes in a Systematic Review, such as eligibility screening, quality assessment, and coding. Kehu Yang is the director of the Evidence‐based Social Sciences Laboratory at Lanzhou University and the author of several Chinese evidence‐based textbooks.

***EGM methods expertise***

Howard White is the lead adviser on evidence mapping and the second coauthor. He has authored a paper on mapping methods used by different agencies that assisted the development of Campbell guidelines and standards for Evidence and Gap Maps and is the author of published and ongoing maps, such as homelessness, disability, violence against children, and youth employment, including the first mega‐map on child welfare and the map of maps in international development.

***Information retrieval expertise***

All authors have previous experience in developing search strategies.

Screening, coding, analysis, and writing will be led by Liping Guo. Overall supervision is provided by Kehu Yang and Howard White.

## DECLARATIONS OF INTEREST

All authors (GLP, LJY, XZ, YKH, RJJ, HXY, CYG, and HW) declared no conflict of interest.

## SOURCES OF SUPPORT

**Internal sources**

• Research on the Theoretical System, International Experience, and Chinese Path of Evidence‐based Social Science, China

The Major Project of the National Social Science Fund of China

**External sources**

• None, China

## Supporting information

Supporting information.
