# Supplementary material for: PROTOCOL: Treatment for depressive disorder among adults: An evidence and gap map of systematic reviews
Source: Campbell Syst Rev. 2023 Mar 5;19(1):e1308. doi: 10.1002/cl2.1308 (PMC9985796; doi:10.1002/cl2.1308)
Supplement: Supplementary file 1 — Supporting information. [file CL2-19-e1308-s001.docx]

# Appendices

## 1 Link to online interactive EGM

Add link at the full report stage
